# Active learning in pharmaceutical 3D printing: a multi-dataset comparison

**DOI:** 10.1007/s13346-026-02077-x

**Published:** 2026-02-24

**Authors:** Moe Elbadawi, Noorul Fathima Abdul Kafoor, Hanxiang Li, Chrystalla Protopapa, Marilena Vlachou, Simon Gaisford

**Affiliations:** 1 School of Biological and Behavioural Sciences, Queen Mary University of London, Mile End Road, London,, E1 4DQ UK; 2 UCL School of Pharmacy, University College London, 29-39 Brunswick Square, London, WC1N 1AX UK; 3 School of Pharmacy, Section of Pharmaceutical Technology, National and Kapodistrian University of Athens, 157 84 Athens, Greece

**Keywords:** Drug development, Artificial intelligence, Computational modelling, In silico, Additive manufacturing, Active machine learning, Sustainability

## Abstract

Machine learning (ML) is expected to accelerate the developments of three-dimensional (3D) printed medicines. Despite ML’s potential, the need for large datasets can hinder progression, as 3D printing remains an emerging pharmaceutical manufacturing technology. This study explores an ML strategy called active learning (AL), which harnesses the benefits of ML whilst applicable with small datasets. AL was tested to predict the printability of three 3D printing datasets: 1437 fused deposition modelling (FDM), 650 vat polymerisation and 297 selective laser sintering (SLS) formulations. The analysis revealed that accuracies of 60% can be achieved when starting with 33 formulations, and subsequent increases in training data size enhances predictive performance. Furthermore, AL was found to achieve 100% predictive accuracy, which is the highest recorded to date for pharmaceutical 3D printing. These initial findings highlight AL’s advantages over traditional ML modelling and showcase its potential to accelerate the development of 3D printing medicines. This research also demonstrates the potential of modelling with small datasets, thereby widening ML’s application in pharmaceutical research.

**Graphical abstract:**

**Figure:**
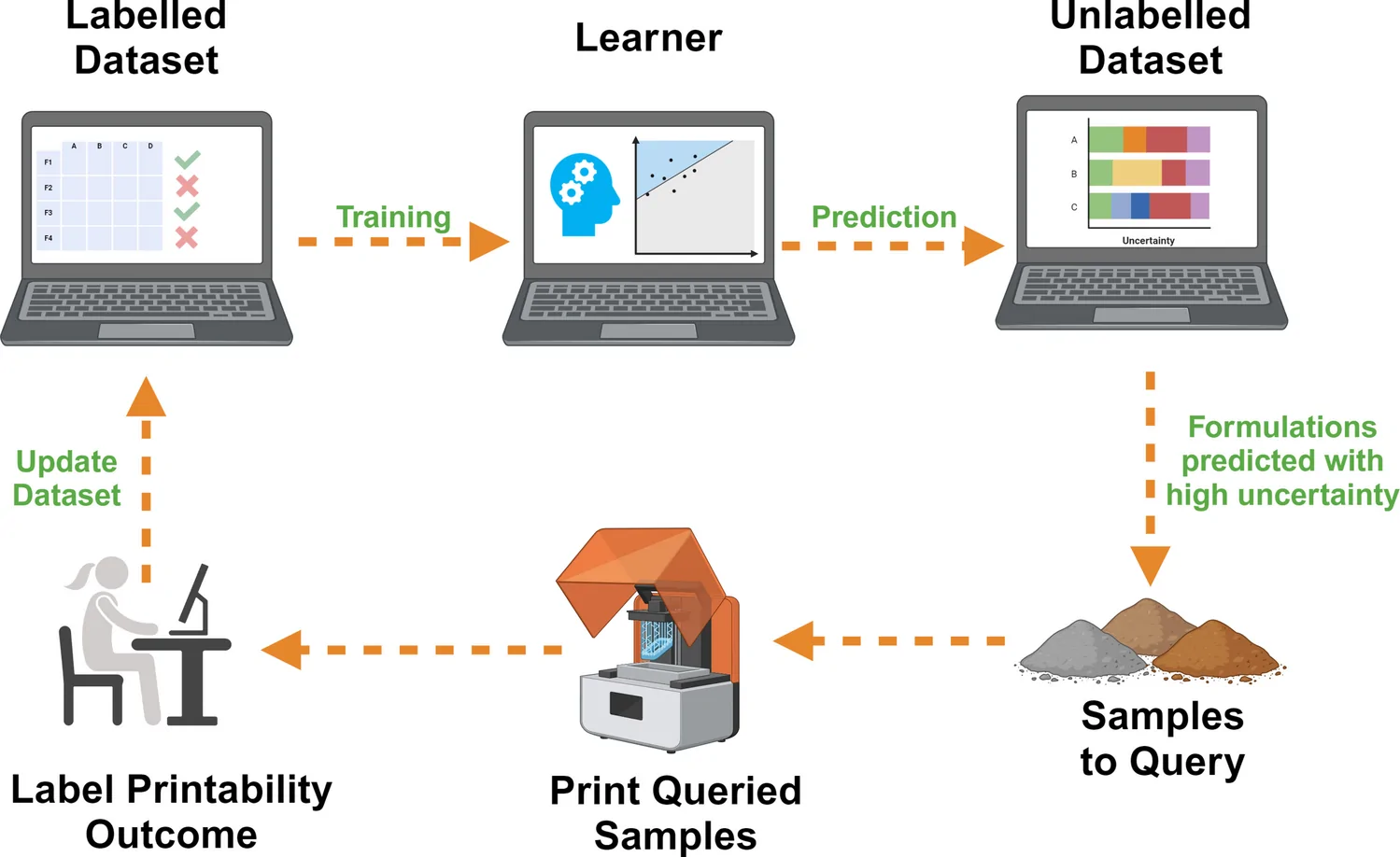

**Supplementary Information:**

The online version contains supplementary material available at https://doi.org/10.1007/s13346-026-02077-x.

## Introduction

Three-dimensional (3D) printing, or additive manufacturing, is a transformative technology in the field of medicine manufacturing [1–7]. The technology produces novel and innovative medicines that have the potential to address unmet clinical needs, particularly in precision medicine [8–13]. Recent examples include Spritam®, a 3D printed medicine for epilepsy, and the recent clearance by the food and drug administration (FDA) for several of Triastek’s investigational new drugs [14, 15]. Briefly, the technology provides an unprecedented level of spatial control, whereby both the interior and exterior structure of a medicine can be precisely designed. This added level of sophistication provides an opportunity to conform to the precision medicine paradigm. 3D printing is advancing our ability to precisely control the dose and drug release profile, such that medicines can be tailored to meet individual needs [16–20].

It is evident that 3D printing has great potential. However, since Spritam’s FDA approval in 2015, there has been little bench-to-bedside translation, which is expected of any nascent technology [21, 22]. Furthermore, as 3D printing can produce near-limitless designs, while indeed a great benefit, means substantial and laborious research is needed to explore all possible designs. Recently, numerous modelling approaches have accelerated developments. This includes physics-based computational models, design of experiments (DoE) and mechanistic models [23–28]. Mixture designs, a type of DoE, can be implemented to consider the formulation space [29–32], but cannot rapidly model it. Like most DoEs, the modelling will function only when all the requested experimental runs have been completed, making the process time-consuming. For example, a mixture design with 10 components (i.e., ingredients) can result in over 1000 experimental runs. Hence, most mixture designs reported in the literature are limited to less than 10 components [33–37].

More recently, machine learning (ML), a subset of artificial intelligence (AI), has been applied to accelerate developments. ML is an emerging modelling technology that has achieved remarkable feats in recent years and transformed the field. In pharmaceutics, ML has been applied across the entire workflow [38, 39], from upstream processes such as predicting drug-excipient compatibility and formulation processability [40–42], to predicting both in vitro and in vivo outcomes [43–45]. Such studies have demonstrated the capabilities of ML modelling in accelerating developments in pharmaceutics. In pharmaceutical 3D printing, research has revealed ML’s utility in tackling numerous processing issues. For quality control, ML has been found to accurately classify the structural quality of 3D printed products in real-time [46, 47]. Moreover, ML has been reported to not only identify the active pharmaceutical ingredient (API) in a formulation but also verify its dose [48]. Furthermore, several studies have demonstrated that ML can predict the 3D printability of formulations and has thus far been applied to fused deposition modelling (FDM), selective laser sintering (SLS), digital light processing and inkjet printing [49–54]. A cutting-edge application of ML in pharmaceutics is the use of generative AI to predict potential formulations [55–59], and by combining AI’s ability with 3D printing, a novel recent study has enabled the generation of de novo 3D printed formulations, and found ML could generate novel formulations that captured the qualities of human-generated formulations [60]. Collectively, these studies have demonstrated the advantages of leveraging ML for pharmaceutical 3D printing.

The benefits of ML over other computational models include the ability to comprehend complex and high dimensional data, interpret different data formats (e.g., numeric, text, images) and super-human comprehension speeds [61–64]. The high dimensionality allows ML to analyse a large number of components simultaneously without needing additional, exhaustive trials. Together, these aspects allow ML to model pharmaceutical processes that have proven challenging with traditional models. Previous work in the field of pharmaceutical 3D printing include predicting the printability and processing temperature, the drug loading and the drug release rate [48, 65, 66]. However, a prominent disadvantage of ML, is the need for large datasets, resulting in researchers and clinicians alike needing to amass numerous formulations to harness the benefits of ML. For example, a recent study achieved prediction accuracies above 90% in predicting printability but required mining the literature for over 900 formulations [66]. This limits the application of ML to the latter stages of development, preventing implementation at the early stages.

The need for large datasets in ML is not exclusive to pharmaceutical sciences. The cost and time associated with generating more data afflicts allied fields and beyond. This has attracted a concerted effort by researchers to develop ML models for small datasets. One particular field of interest is active ML or active learning (AL). It is termed ‘active’ because it actively learns as it receives more data, which helps to improve its predictive performance. AL models continuously interact with researchers throughout the experimental process and, remarkably, can dictate the experiments conducted for its learning. AL’s application in guiding researchers through the experimental space allows AL to not only make predictions but also alleviate the decision making of which experiments to test next. This makes AL useful in the early stages of development, as it can be trained on a small dataset yet tasked with predicting outcomes for much larger datasets. Simply put, the primary goal of AL is to map the experimental space with as little experiments as possible. In molecular medicine, several studies have found that AL can drastically reduce the number of experiments conducted [67–75]. In addition to its accuracy, the speed at which AL performs has prompted researchers to incorporate AL into automated laboratory robots, such that AL guides the robotic platform in which samples to test, rather than testing all samples [76].

To that end, we investigated the potential of AL for accelerating developments in pharmaceutical 3D printing. To examine its versatility, we applied AL retrospectively to three distinct datasets (fused deposition modelling (FDM), vat polymerisation and selective laser sintering (SLS)), where each one was comprised of different sample sizes, numbers of components and ratios of printable to non-printable formulations. It is worth noting that this was the first study in predicting the printability of vat polymerisation pharmaceutical formulations using ML. We examined the effects of starting sample size and the effects of three different machine learners. The principles of AL are provided in the supplementary (**Section S1**).

## Experimental procedure

### Data collection

All three datasets were a combination of both in-house and literature-extracted formulations. The structure of the datasets in essence contained the name of the formulation, the proportion of the individual components and their printability. Printability was determined based on the success of a printing process without visible defects. Further information can be found in the supplementary (**Section S2**).

This structured approach ensures that each formulation is accurately labelled based on explicit descriptions or inferred from the outcomes and focus of the study. Table 1 presents an example of the dataset structure.

**Table 1:** Representation of the dataset structure

Source	Sample Name	Excipient_1_ (w/w%)	Excipient_2_ (w/w%)	…	Drug_a_ (w/w%)	Drug_b_ (w/w%)	Printability
10.xxx/xxx	F1	90	0	**…**	0	10	Yes
10.xxx/xxx	F2	0	80	**…**	20	0	No
…	**…**	**…**	**…**	**…**	**…**	**…**	**…**
In-house	S25	45	35	**…**	20	0	Yes

### FDM

The FDM dataset is a combination of the in-house and literature-extracted dataset previously used for ML modelling [65, 66]. Briefly, the dataset comprised of the formulations and the individual component proportion, and the formulation’s printability.

### SLS

PubMed, Google Scholar, and Web of Science were used to search for articles published in English using the terms “SLS 3D printing”, or “selective laser sintering”, along with the keyword “drug”, wherein articles based on diode SLS printers were selected. The search dates were set to between August 1, 2017, and April 2, 2024. The formulation composition and printability were recorded.

### Vat polymerisation

PubMed, Google Scholar, and Web of Science were used to search for articles published in English using the terms “SLA 3D printing” or “stereolithography” with the keyword “drug”. The search dates were set to between January 1, 2016, and March 3, 2024. The formulation composition and printability were recorded.

### Feature generation

The input features for model training consisted of the formulation's composition—each material within a formulation was considered a distinct feature. The proportion of each component, measured in weight-by-weight percentage (w/w%), serves as the input value for that feature. Hence, for each formulation analysed, the model includes all its ingredients as inputs, alongside their respective proportions. In cases where a formulation lacks a component present in other formulations, a value of zero is assigned for that component's feature. The consequent feature set generated has been previously referred to as ‘Material Name’ [77].

### Software & hardware

The subsequent model development and metric evaluation were performed using the programming language Python (v 3.7.6) using the machine learning library *scikit-learn* (v 1.0.2), also referred to as *sklearn*. A standard PC was used for programming the pipeline (MacBook Pro 2017; Processor: 3.1 GHz Dual-Core Intel Core i5; RAM: 8 GB 2133 MHz LPDDR3).

### Active learning modelling

**Fig. 1:**
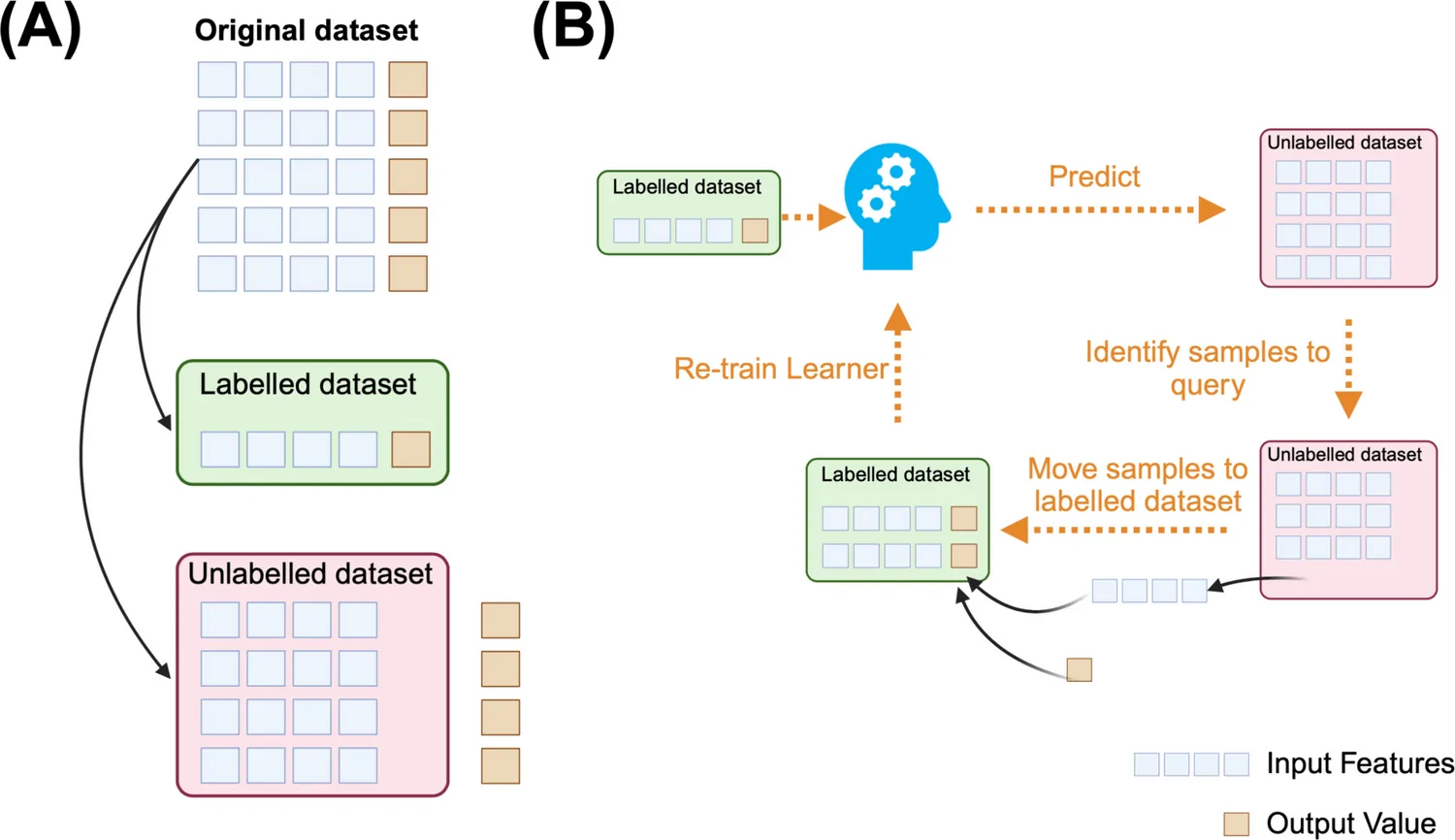
Illustration of the querying cycle. **(A)** First, the original dataset was randomly divided into a labelled and unlabelled dataset. **(B)** The learners were trained on an initial starting sample set and during each query cycle, the labelled dataset was updated with formulations identified with the highest predictive uncertainty

Three classification learners were used for the AL modelling: random forest (RF), artificial neural networks (ANN) and logistic regression (LR). All learners were initialised from their *scikit-learn* library using their default parameters. The ‘.predict_proba’ method was used to retrieve the predicted probability estimates. The *numpy* library (v 1.20.3) was used to implement Eq. (1). In essence, the top five formulations with the highest uncertainty (i.e., lowest confidence) were prioritised and subsequently queried by the learners. This uncertainty method is referred to as the least confidence method. The effect of the starting sample size was also investigated, where a starting formulation size of 5, 10 and 20% was investigated, where data was selected at random using the *train_test_split* function in *sklearn*. To ensure reproducibility, each analysis was conducted five times with distinct random states set to 1, 5, 10, 50, and 100. The learners were trained on both the inputs and output for these starting dataset sizes, representing the labelled data. It was then tasked with predicting the outcomes for the remaining unlabelled data, constituting 95%, 90%, and 80% of the sample size, respectively, without access to their actual output values (i.e., printability). After the prediction, 5 formulations were transferred from the unlabelled to the labelled dataset, where their output values were also added. This process continued until 80% of the overall data was used for training the learners. A schematic of the process is illustrated in Fig. 1.

### Data evaluation

For classification, the primary metrics were balanced accuracy and Matthew’s correlation coefficient (MCC), and were implemented using the ‘*balanced_accuracy_score*’ and ‘*matthews_corrcoef*’ functions in *sklearn*. These were defined as:

1 $$Balanced\;Accuracy=\frac12\left(\frac{TP}{TP+FN}+\frac{TN}{TN+FP}\right)$$

2 $$MCC= \frac{TP \times TN-FP \times FN}{\sqrt{\left(TP+FP\right)\left(TP+FN\right)\left(TN+FP\right)\left(TN+FN\right)}}$$

where TP is the true positive, TN is the true negative, FP is the false positive and FN is the false negative. The mean and standard deviation of the five analyses were recorded. For the external dataset, we have also employed Brier’s Score and the F1, of which their implementation can be found in refs [46] and [65], respectively.

## Results

All three datasets were prepared for our AL simulations were comprised of formulations generated both in-house and from the literature, providing a diverse and comprehensive set of formulations for the simulations. The distinct datasets present AL with their own characteristics, as detailed in the next section, allowing us to ‘stress-test’ AL’s ability to generalise its learning, rather than simply learning our idiosyncrasies in preparing 3D printing formulations.

### Exploratory data analysis (EDA)

**Fig. 2:**
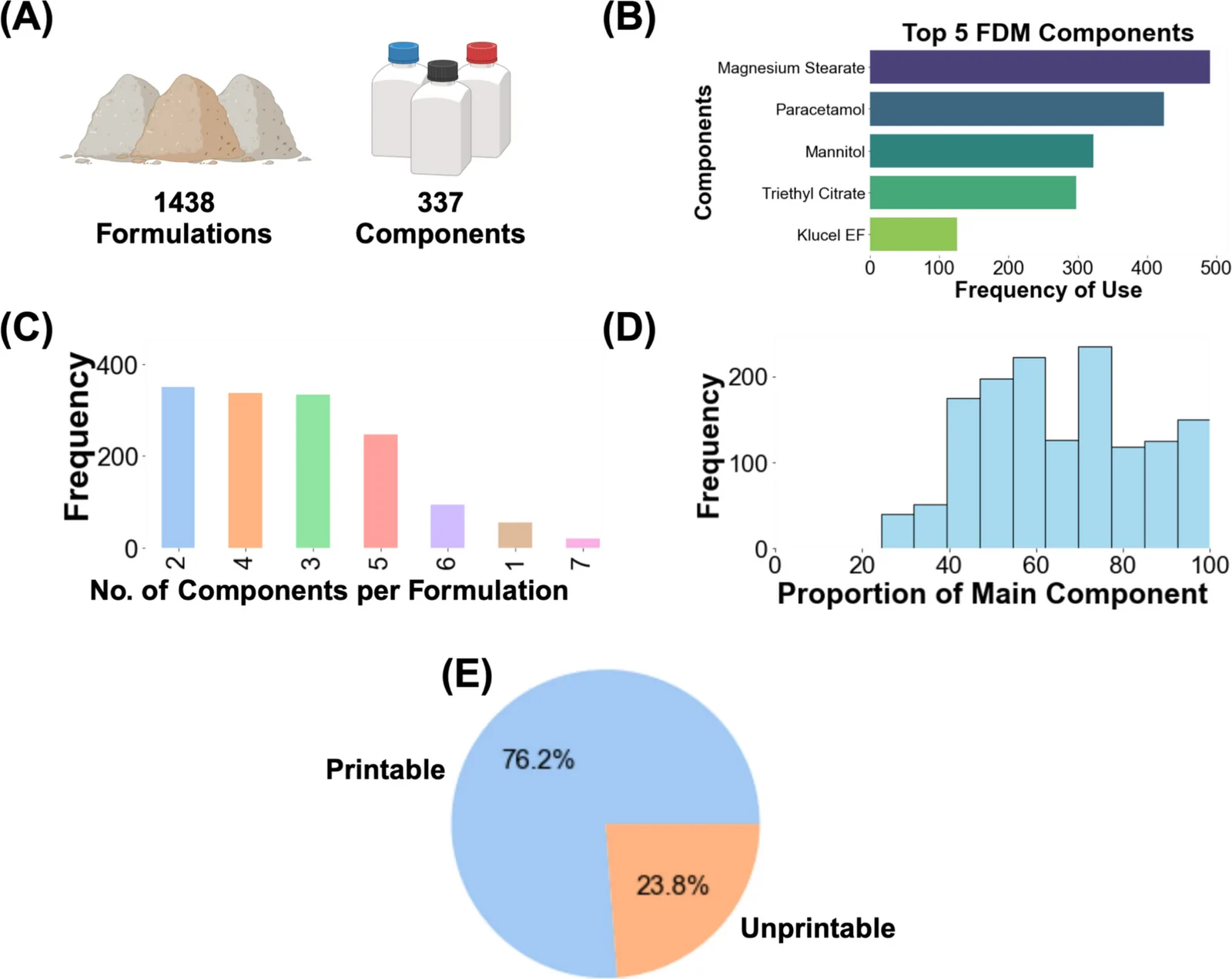
Insight into the FDM dataset. (**A**) Total number of formulations and components. (**B**) Top 5 used components. (**C**) Number of components per formulation. (**D**) The proportion of the main component (w/w%). (**E**) The ratio between printable and unprintable formulations

FDM is amongst the most widely studied 3D printing technique in pharmaceutics and therefore had the largest dataset in terms of both formulations and components, with 1,438 formulations from 336 components (Fig. 2). The top five components within the dataset were magnesium stearate (n = 491; 34%), paracetamol (n = 424; 30%), mannitol (n = 323; 22%), triethyl citrate (n = 298; 21%) and Klucel® EF (n = 126; 9%). The number of components per formulation ranged between 1 to 7, with 2–4 components per formulation being of comparable frequency. A pharmaceutical formulation comprising of an excipient and API is plausible, hence a two-component formulation was frequent. The addition of a dry lubricant (e.g., magnesium stearate) and/or a plasticiser (e.g., mannitol) facilitates the process. The proportion of the main component, typically a polymer matrix, varied, with the mode being 70—75 w/w%. Furthermore, the ratio of printable formulations to non-printable formulations was 76.2:23.8, which was considered imbalanced.

**Fig. 3:**
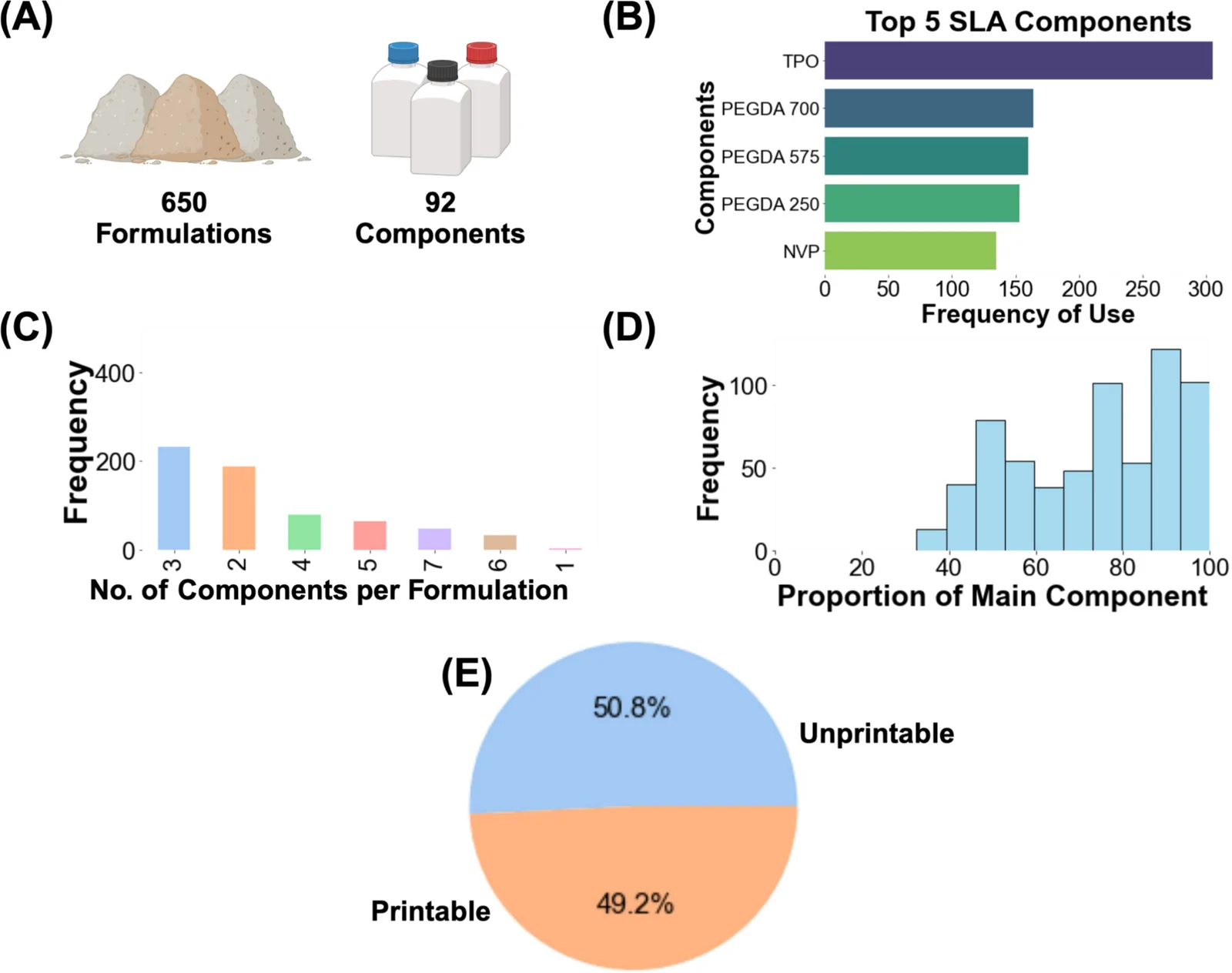
Insight into the vat polymerisation dataset. (**A**) Total number of formulations and components. (**B**) Top 5 used components. (**C**) Number of components per formulation. (**D**) The proportion of the main component (w/w%). (**E**) The ratio between printable and unprintable formulations

Vat polymerisation formulation development is not as established as FDM, hence the dataset comprised of fewer formulations and components, which were 650 and 92, respectively (Fig. 3). Furthermore, vat polymerisation is limited to photopolymer-based formulations, meaning not all excipients are FDA-approved. This partly explains the lower formulation number. The five most common components in the vat polymerisation dataset were diphenyl(2,4,6-trimethylbenzoyl)phosphine oxide (TPO; n = 305; 47%), polyethylene glycol diacrylate 700 (PEGDA 700; n = 164; 25%), PEGDA 575 (n = 160; 25%), PEGDA 250 (n = 153; 24%) and N-vinylpyrrolidone (n = 135; 21%). Vat polymerisation requires a photopolymer and photoinitiator to work, and except for the photoinitiator TPO, the top five components were photopolymers. PEGDAs are a popular choice of photopolymers for pharmaceutical vat polymerisation due to their biocompatibility, if appropriately processed [78, 79]. The number of components per formulation ranged from 1–7 components, with the most popular being 3 components. The key components in an vat polymerisation formulation are a photopolymer, a photoinitiator and an API. In some cases, a plasticiser or a diluent is added to adjust the viscosity, resulting in a total of four components [80, 81]. The majority of vat polymerisation formulations contained one major component, as evidenced by the histogram, with approximately 50% of formulations containing a component comprising over 70 w/w%. The ratio of printable to non-printable vat polymerisation formulations (49.2:50.8) was considered balanced.

**Fig. 4:**
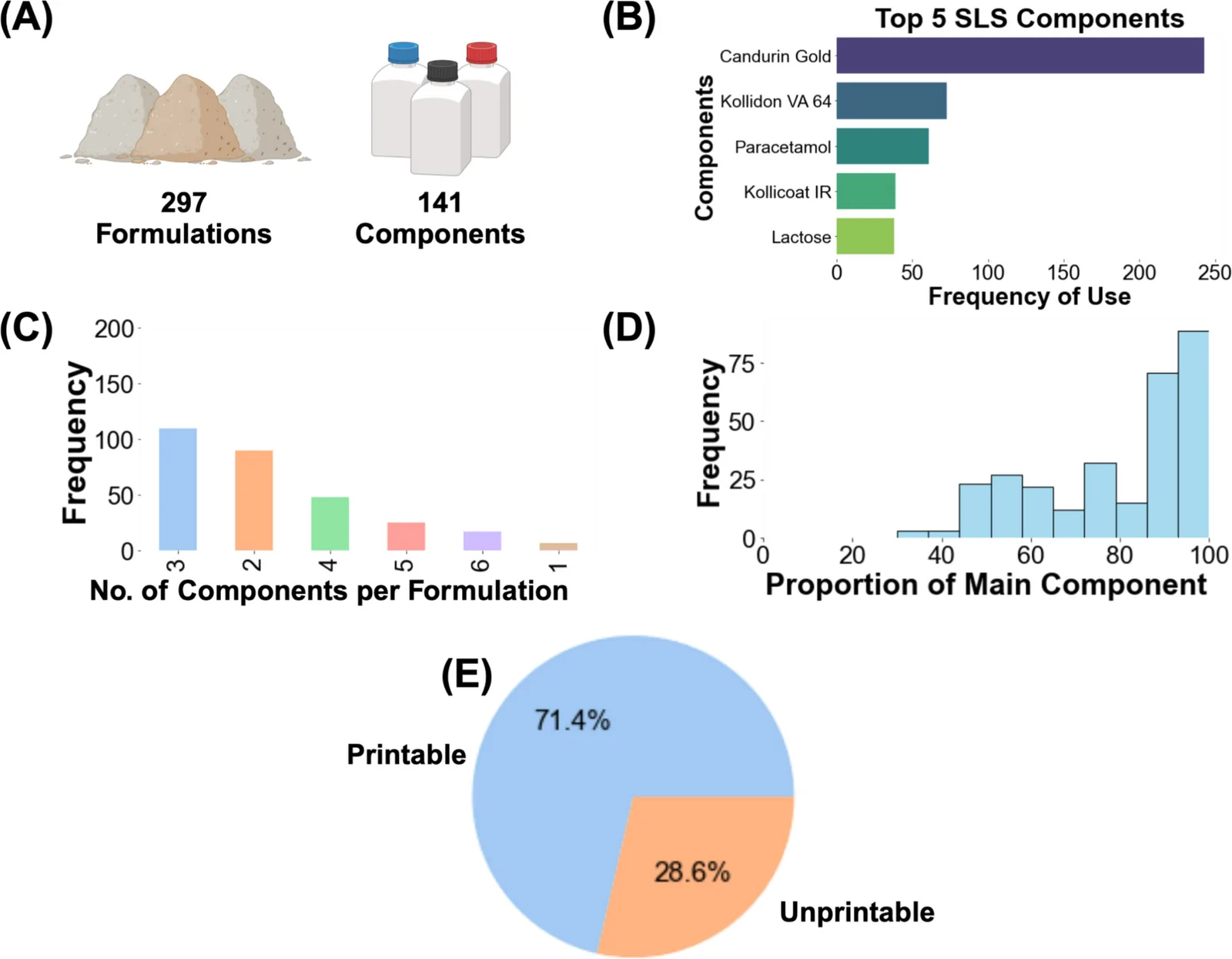
Insight into the SLS dataset. (**A**) Total number of formulations and components. (**B**) Top 5 used components. (**C**) Number of components per formulation. (**D**) The proportion of the main component (w/w%). (**E**) The ratio between printable and unprintable formulations

SLS is the least studied technology in pharmaceutics of the three investigated, mainly driven by a few research groups. Hence, the total number of formulations is notably smaller than both FDM and vat polymerisation, with 297 formulations from 141 components (Fig. 4). SLS is compatible with a range of pharmaceutical grade excipients, such as Kollicoat® IR and Eudragit® L100-55, providing researchers with a broader option of components to select from. The five most popular components within the dataset were Candurin® gold (n = 243; 82%), Kollidon® VA 64 (n = 73; 25%), paracetamol (n = 61; 21%), Kollicoat® IR (n = 39; 13%) and lactose monohydrate (n = 38; 13%). 82% of the formulations contained Candurin® gold, which is used as a photoabsorber. For diode-based SLS printers, a photoabsorber is needed to facilitate sintering, and to date, is considered a necessary component in diode-based SLS formulations. The number of components per formulation ranged from 1–6, with the most frequent being 3 components per formulation. A typical SLS formulation contains a polymer, a photoabsorber and an API. Similar to vat polymerisation, the majority of formulations were comprised of one major component, with over 50% of formulations containing at least 80 w/w% of one component. For printability, the ratio was 71.4:28.6 in favour of printable, and thus, the dataset was notably imbalanced.

The EDA revealed several differences between the three datasets: Two datasets presented with an imbalanced printability ratio. Training learners on an imbalanced dataset could lead to bias in its prediction towards the majority class (i.e., the more frequent class) [82]. Other differences included the dataset size, with SLS possessing the smallest and FDM being 4.9-folds larger, with respect to formulation sample size. The number of components also greatly varied, with vat polymerisation possessing the smallest and FDM possessing ~ 3.7 times more. Further differences included formulation characteristics, including the component proportions. Such diversity amongst the three datasets will allow us to observe how AL can adapt accordingly. In particular, as will be discussed below, AL’s ability to handle imbalanced datasets was much anticipated.

### AL analyses

AL was applied to all three datasets where the size of the starting dataset was explored at 5%, 10% and 20%. At each iteration, 5 formulations were queried, and the tests were allowed to run until 80% of the training data has been used. Three different learners with different learning abilities, LR, RF and ANN, were explored. The learners were not optimised and were used in the default setting, as it would be difficult to know a priori what the optimal parameters for each learner are. All analyses were repeated 5 times for reproducibility, with different starting formulation samples selected and no stratification applied.

For performance assessment, balanced accuracy was chosen as the primary metric. In contrast to metrics like standard accuracy and F1 score, which can be misleading with imbalanced datasets, balanced accuracy is suitable for the FDM and SLS datasets. Metrics such as accuracy can yield overly optimistic values; for example, if a dataset has 75 out of 100 formulations labelled as “printable,” a model predicting all formulations as “printable” would still achieve 75% accuracy without learning to distinguish between classes. Other metrics better suited to imbalanced datasets include MCC, area under the receiver operating characteristic curve, and Cohen’s Kappa. These metrics more effectively measure if a model is learning patterns beyond majority-class predictions. However, these metrics require the presence of both majority and minority class samples in each iteration, or their calculation becomes undefined, resulting in an error. We found frequent instances in the FDM and SLS datasets where the active learner queried only ‘printable’ formulations, resulting in no negative samples, leading to a lack of true or false negatives and causing calculation errors.

In our application, balanced accuracy proved to be the most appropriate choice. Like accuracy, it operates on a 0–100% scale, but accounts for correct classification of both majority and minority classes. No calculation errors will occur despite the absence of the minority class in iterations. If a query iteration includes both classes, a model correctly predicting only the majority class will have a balanced accuracy of 50%, reflecting its balanced nature. In this instance, values above 50% indicate that the model was able to predict both the majority and minority class and values below 50% indicate the model was struggling to differentiate between them.

**Fig. 5:**
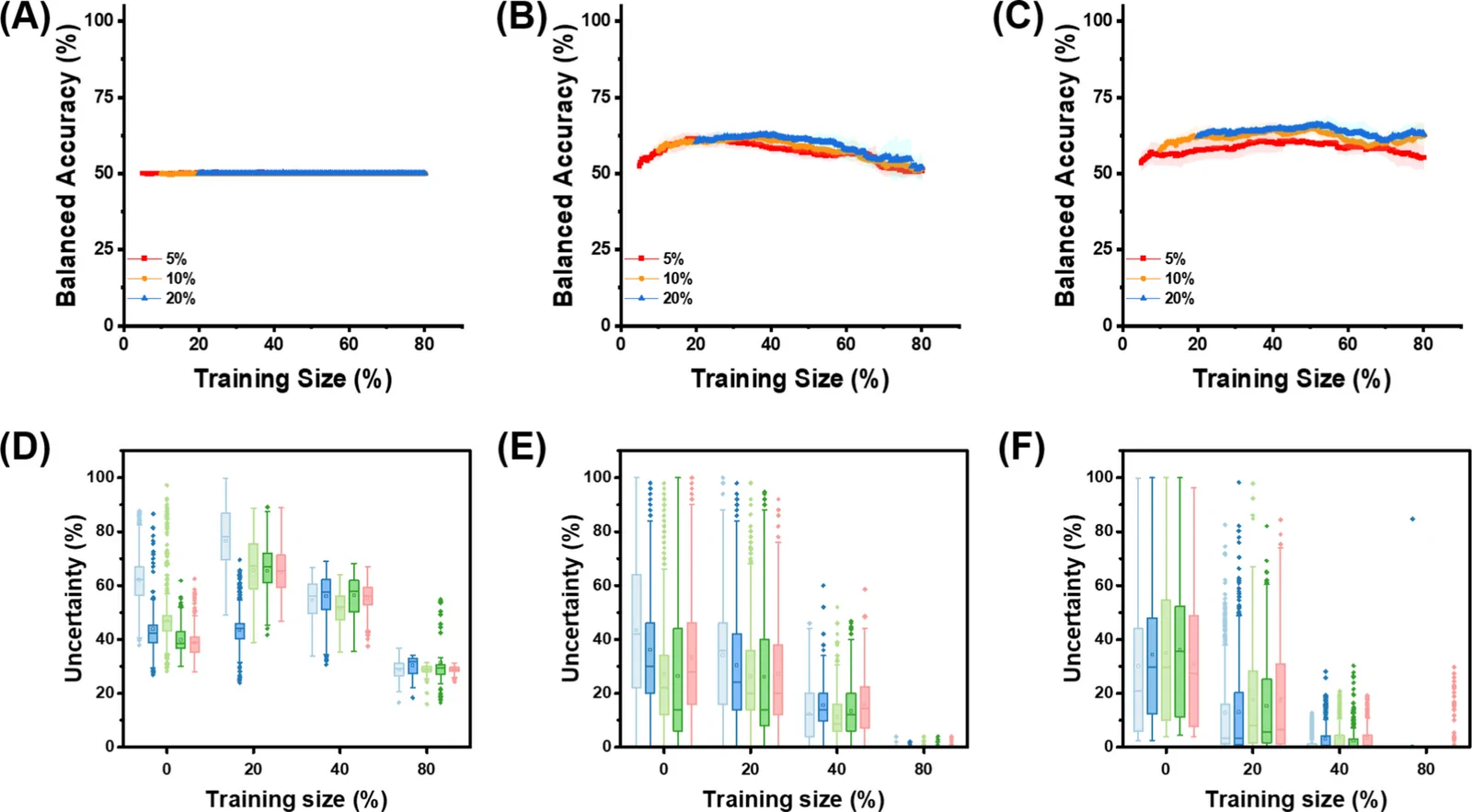
Balanced accuracy scores (**A-C**) and the uncertainty (**D-F**) for predicting FDM printability using (A, D) LR, (B, E) RF and (C, F) ANN. The shaded region represents the standard deviation. The distribution of prediction uncertainties at 5 random states, at 0%, 20%, 40% and 80% training size is shown

Starting with FDM, all three learners evidently struggled (Fig. 5 (A) – (B)). For example, LR remained at 50% balanced accuracy, irrespective of the starting sample size, inferring that the learner was unable to differentiate between the two classes. RF was found to initially improve with increasing training size but then decreased towards 50%. ANN was found to be the better performer here, where the balanced accuracy was constantly above 50%, and did not plummet like RF towards the end. The balanced accuracy for ANN with starting sample sizes of 5%, 10% and 20% were 55.16 ± 3.96, 62.49 ± 3.45 and 62.87 ± 3.09%, respectively, at the end of the simulation. The corresponding MCC scores can be found in Figure S2. The uncertainty was also inspected at the early stages of the AL cycle, from 0–40% of the training size, and then once more at the end (i.e., 80% training size) (Fig. 5 (D) – (F)).Surprisingly, uncertainty was found to decrease in all learners with increasing training size, suggesting that learners became more confident in their predictions, irrespective of changes in model balanced accuracy.

**Fig. 6:**
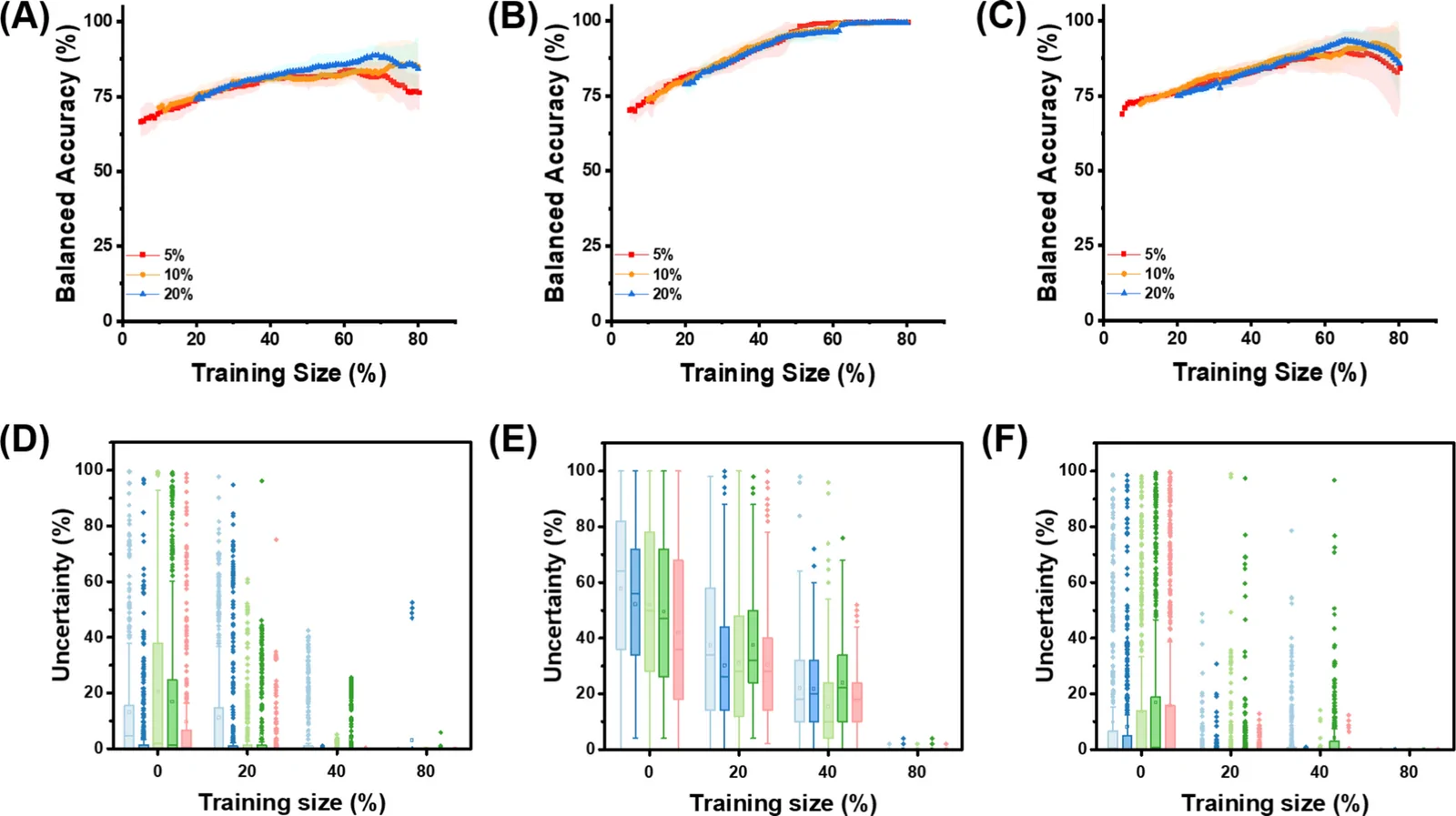
Balanced accuracy scores (**A-C**) and the uncertainty (**D-F**) for predicting vat polymerisation printability using (A, D) LR, (B, E) RF and (C, F) ANN. The shaded region represents the standard deviation. The distribution of prediction uncertainties at 5 random states, at 0%, 20%, 40% and 80% training size is shown

For vat polymerisation, noticeably higher balanced accuracies (Fig. 6) and MCC scores (Figure S3) were achieved. Other characteristics included starting off at higher accuracies, continually increasing as more training sample sizes were selected, and in the case of RF, reaching 100% accuracy. Furthermore, and in contrast to the FDM analysis, the uncertainty for all three learners decreased to a mean of 0. Hence, the analysis suggests that in balanced datasets, AL performs well, and uncertainty can be used as a measure of prediction performance if applied prospectively. Furthermore, given that balanced accuracy was above 70% from the outset, AL can be applied at early stages of vat polymerisation formulation, and with small datasets.

**Fig. 7:**
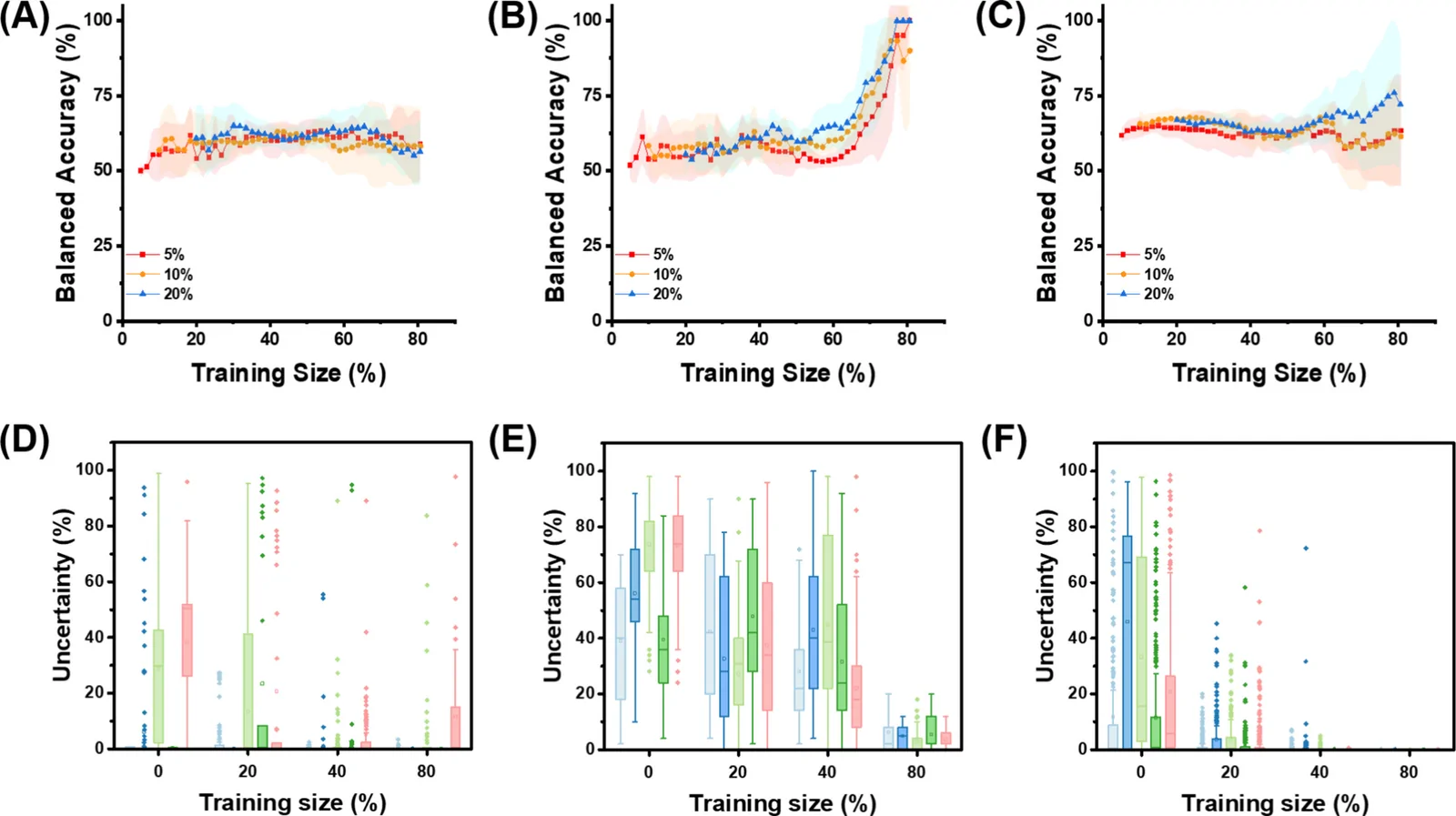
Balanced accuracy scores (**A-C**) and the uncertainty (**D-F**) for predicting SLS printability using (A, D) LR, (B, E) RF and (C, F) ANN. The shaded region represents the standard deviation. The distribution of prediction uncertainties at 5 random states, at 0%, 20%, 40% and 80% training size is shown

For the SLS dataset, LR mean balanced accuracy began at 50% and marginally increased before plateauing at approximately 60% (Fig. 7). At approximately 70% of the training size, a small decrease was observed. The final accuracies for the starting sample size of 5%, 10% and 20% were 58.76 ± 11.37%, 58.26 ± 12.49% and 56.40 ± 10.75%, respectively. For RF, the accuracy was initially comparable to that of LR, but increased at ~ 64% of the training dataset size for all three starting sample sizes. The final accuracies were 100.00 ± 0.00%, 90.00 ± 22.36% and 100.00 ± 0.00% for 5%, 10% and 20% starting sample sizes, respectively. In contrast, ANN exhibited a higher mean accuracy at the outset, ranging from 61.78 to 66.95%. For 5% and 10% starting sample sizes, the mean accuracy oscillated in this region. When starting with 20% of the data, a noticeable increase was observed when ~ 52% of the data was used. However, this was not found to be significant due to the large standard deviations observed across all three starting sample sizes. The MCC scores for the SLS dataset presented with similar trajectories and can be found in **Figure S4**. Furthermore, and in concert with the other two datasets, the uncertainty decreased as the learners progressed through the AL cycle.

### Weighting the uncertainty

From the initial AL analysis, it was evident that AL was effective for the vat polymerisation dataset but produced inconsistent performances for the FDM and SLS datasets. One possible explanation was that imbalance in the datasets hindered AL performance, with learners prioritised the majority class and consequently were ineffective in predicting the minority class. This is more pronounced when the initial training samples all belong to the majority class, which invariably leads to learners assuming the remaining samples belong to one class. To address this issue for the FDM and SLS datasets, a weighting factor was applied to the uncertainties of the minority class in each iteration, increasing their priority for being queried. The uncertainties were multiplied by the three weighting factors: 1.25, 1.5 and 2x. Previous analyses revealed no difference in balanced accuracy between the starting sample sizes, hence analyses going forward had an initial starting size of 5%.

**Fig. 8:**
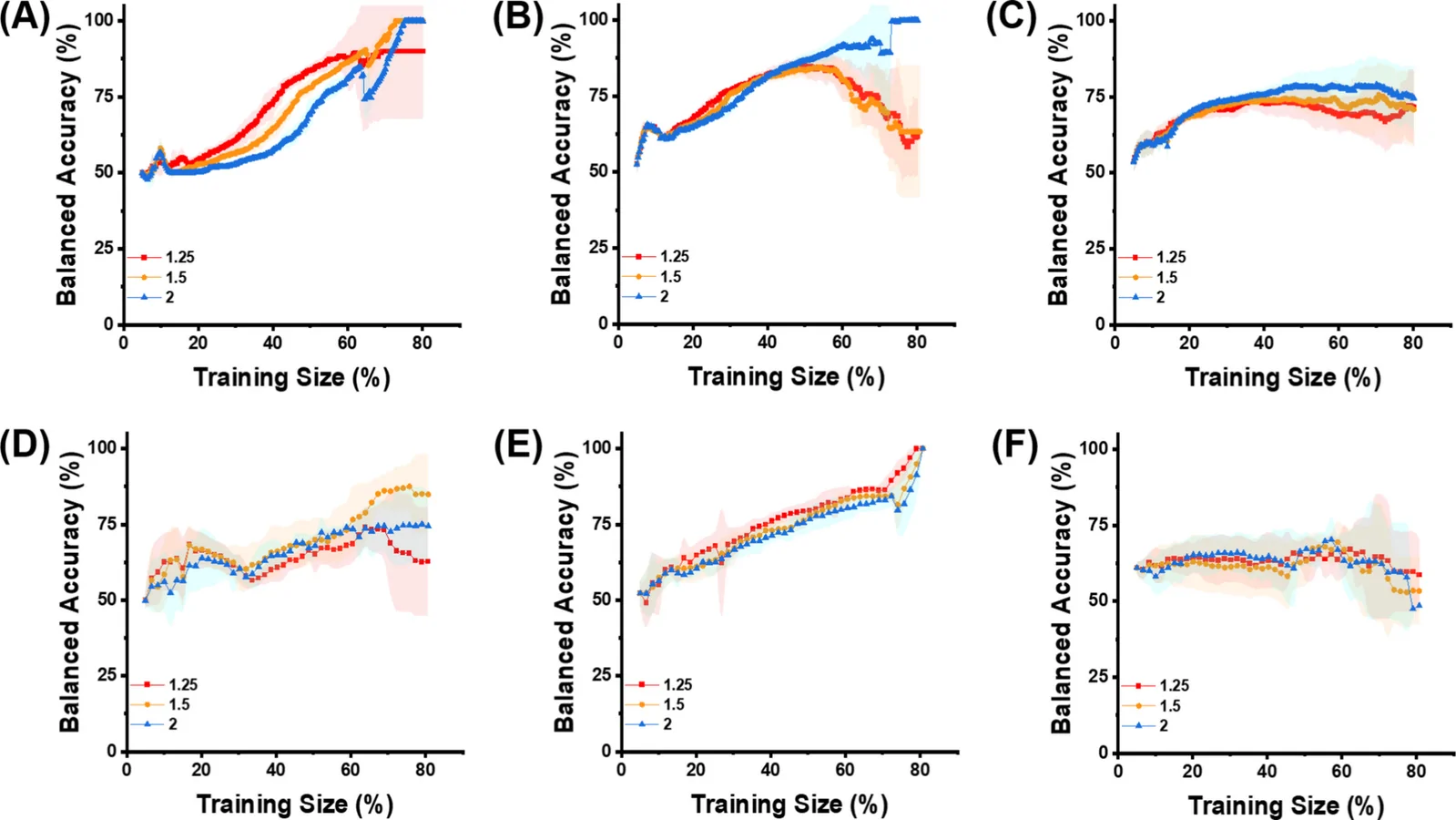
The effect of weighting the minority class for the (**A-C**) FDM and (**D-F**) SLS dataset using (A, D) LR, (B, E) RF and (C, F) ANN. The shaded region represents the standard deviation

For the FDM dataset, a significant improvement in the balanced accuracy was observed when using LR as the learner (Fig. 8 (A) – (C)). The initial mean accuracy was 50%, which subsequently increased as more samples were queried, depending on the weighted factor. A 1.25 × weighting factor increased accuracy between ~ 20% and ~ 68% training dataset size, stabilising at a mean accuracy of 90%. Increasing weighting to 1.5 and 2.0 × required more training data to observe an increase in accuracy, but both culminated in accuracy of 100% at 73% and 76% of the training size, respectively. For RF, the balanced accuracy reached 100% when applying a 2 × weighting factor. In contrast, applying a 1.25 and 1.5 × weighting factor resulted in an initial increase in accuracy until ~ 56% of the training data was used, followed by a steady decrease to ~ 62%. For ANN, the accuracy steadily increased to when ~ 42% of the training data was used, plateauing at a mean accuracy of ~ 73%. Therefore, it is clear that applying a weighting factor to the minority uncertainty values largely had a positive effect on AL performance for the FDM dataset.

For the SLS dataset, improvements were seen with LR (Fig. 8 (D) – (F)). Applying a weighted factor of 1.5 × resulted in accuracy of 69.34 ± 7.30% when ~ 54% of the training size was used, increasing to 84.87 ± 13.34% at the end of the analysis, which was a significant improvement to the unweighted accuracies (Fig. 7 (A)). Applying the weighted factors to the RF learner also impacted its performance, and accuracy continuously increased towards 100%. For ANN, the weighting factor was found not to have a significant impact on performance, until ~ 58% of the training data was reached, after which, a decrease in performance was observed. Thus, the overall performance was hindered compared to the unweighted strategy (Fig. 7 (C)).

### Ensemble learning

**Fig. 9:**
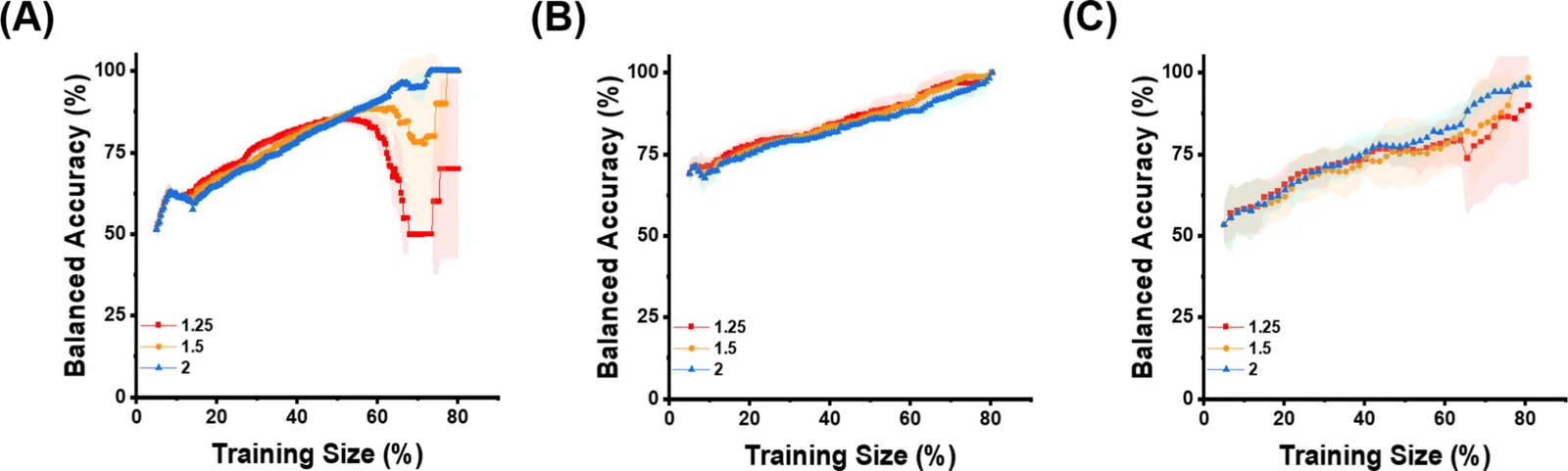
The results of the ensemble learning for (**A**) FDM, (**B**) vat polymerisation and (**C**) SLS dataset. The shaded region represents the standard deviation

The two previous analyses highlighted one interesting finding; there was no obvious optimal learner. LR performed well for the vat polymerisation dataset, and for both the FDM and SLS datasets when the uncertainty of the minority class was weighted. However, this improved performance seldom occurred at the early stages of the AL cycle. RF generally performed well, and the FDM dataset benefited from applying a 2 × factor to the uncertainty values of the minority class. Contrastingly, ANN appeared less affected by the weighting factors when applied to the FDM dataset than RF, but struggled with the SLS dataset in comparison. Thus, it was evident that the dataset favoured some learners over others. This led to the next investigation, which was combining the querying of all three learners. Here, the uncertainty was averaged across all three learners, and samples with the highest average uncertainty were prioritised. Hence, this was not a query by committee strategy, but rather an ensemble of the uncertainties of LR, RF and ANN. The strategy was applied to all three datasets, including the vat polymerisation dataset to determine whether it could significantly affect its performance compared to the previous strategy (Fig. 9).

For the FDM dataset, the best performance was observed when applying a 2 × weighting to the uncertainty; balanced accuracy continuously grew and reached 100%. Furthermore, the small standard deviation revealed that the performance was highly reproducible. For the vat polymerisation dataset, the initial accuracy of ~ 69% increased to 100%, with no differences observed by the different weighting factors. Similarly, for the SLS dataset, the accuracy increased from the outset as the learners progressed through the AL cycle. The final accuracies for the 1.25, 1.5 and 2 × weighted factors were 89.82 ± 22.26%, 98.40 ± 1.57% and 96.33 ± 3.70%, respectively. Hence, increasing the weighted factor to either 1.5 or 2 × yielded higher and more stable accuracies.

### Case studies

**Fig. 10:**
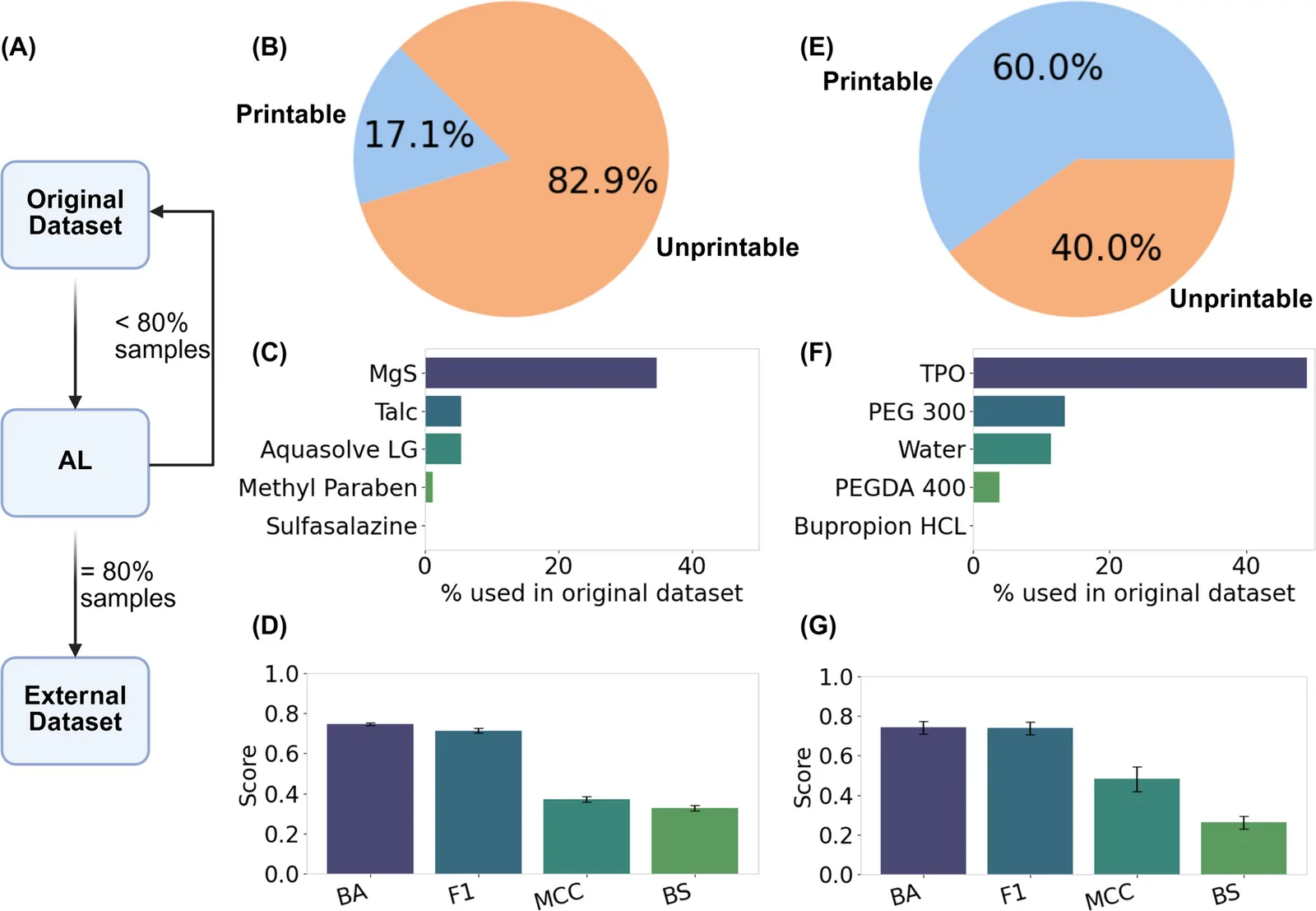
(**A**) A schematic of the AL process for both Case Studies. Using the optimal methodology (Fig. 9), the AL was subsequently tasked with predicting the external FDM and vat polymerisation datasets. (**B**) – (**D**) are the ratio of printability, ingredients and their respective frequency in the original dataset and the metrics for the external FDM dataset. (**E**) – (**G**) are the ratio of printability, ingredients and their respective frequency in the original dataset and the metrics for the external vat polymerisation dataset. Note, the metrics were normalised in (**D**) & (**G**) to between 0–1. (BA—balanced accuracy; MCC—Matthew’s Correlation Coefficient; BS – Brier’s score)

The above AL approach is considered interpolation, since the ML technique is searching within the same formulation space and is sampling all aspects of the formulation space as it decides which samples to test next. To ‘stress-test’ the AL, we applied it to external datasets, where the formulations were unique and unseen to the AL (Fig. 10 (**A**)). We conducted two case studies, one for FDM and one for vat polymerisation. In both cases, a mixture DoE was used to generate a systematic experimental run for APIs not present in the existing datasets, which were then tested and their printability were recorded. Subsequently, we took the respective trained AL and applied it to predicting the printability in both external dataset. The experimental protocol for both case studies, including printing protocol, are included in the supplementary document.

**Case Study 1** focused on the FDM printing of sulfasalazine, which was an ongoing project for the authors, and to the authors’ knowledge, has not been printed using FDM. The external FDM dataset contained 83% unprintable formulations (Fig. 10 (**B**)), thus making it the opposite of the original FDM dataset (Fig. 2). Furthermore, of the 5 ingredients in the mixture design, 4 were available in the original dataset and one was totally new, which was the API sulfasalazine. Of the 4 already found in the original dataset, their usage varied, ranging from magnesium stearate used 34% in the original dataset, to methyl paraben NF grade used 1% (Fig. 10 (C)). Using the best performing AL strategy (i.e., up to 80% of the original dataset, ensemble learning and a weighted factor of 2x), the balanced accuracy, weighted f1, MCC and Brier’s score were 74.6 ± 0.8, 71.4 ± 1.2, 37.3 ± 1.3 and 32.7 ± 1.3, respectively (Fig. 10 (D)). These metrics suggest that while the model performed better than random guessing, the Brier’s score indicated its confidence was low [46]. For **Case Study 2**, the dataset contained unique vat polymerisation formulation. The printability was 60% printable (Fig. 10 (**E**)), thus, more imbalanced than the vat polymerisation original dataset (Fig. 3). The formulations also comprised five ingredients of which 4 already existed in the training dataset and one was novel. Of the already existing, their proportion in the original dataset varied from TPO (the photoinitiator) being used 47% to PEGDA 400 used 4% in the training dataset (Fig. 10 (F)). Using the best performing AL strategy, the balanced accuracy, weighted f1, MCC and Brier’s score were 74.2 ± 3.1, 73.8 ± 3.2, 48.3 ± 6.3 and 26.2 ± 3.2, respectively (Fig. 10 (G)). These metrics suggest that while the model performed better than random guessing, its confidence was moderate. Therefore, both Case Studies demonstrated that AL was capable of accurately predicting the printability in external databases, with formulations not found within the original training dataset, albeit a decrease in accuracy compared to the original dataset. Furthermore, it also demonstrates that the AL strategy can be transferred to formulations containing ingredients not previously found in the training dataset.

## Discussion

AL has the potential to become a key computational modelling technique in pharmaceutical research, harnessing the advantages of ML whilst applicable to datasets where only a small amount of processability is known (e.g., printability). This is particularly beneficial for emerging technologies like 3D printing, helping to accelerate their development and integration into the sector at the early stages of development. Furthermore, as governmental policies change, like moving towards carbon neutrality, enabling technologies that can improve the agility of 3D printing will be desired [83]. As mentioned, the aim was to initiate our understanding of prospectively applying AL at the early stages of formulation development and the results herein are promising.

The multi-dataset analysis revealed that additional processing and combining the learning of multiple ML techniques increases model performance (Fig. 8 and Fig. 9). In doing so, AL can achieve high accuracies for predicting printability whilst utilising a fraction of the data previously used in ML [65, 84, 85]. A key finding from this study was that the formulation space can negatively impact AL performance, as learners struggled with formulation spaces containing an imbalance between printable and unprintable formulations. This was not only an issue for the initial training samples but was an issue in all subsequent training iterations. In other words, one can begin AL by training it with both printable and unprintable formulations, however, if the remaining formulation space is imbalanced then it will impact the learner’s performance. While the ensemble learning combined with weighting the uncertainty of the minority class mitigated the negative affect of an imbalanced dataset. Additional work is needed to understand what other types of complexities can hinder AL performance before the technology is widely adopted in formulation development.

In addition to the highly accurate predictions, the ability of the learners to navigate through the formulation space independently, without human intervention, and determine which samples to test, is a welcomed benefit, as it removes the need for decision making by the researcher, allowing them to redirect their efforts to other tasks. There is also the possibility of integrating AL into hardware like 3D printers and other robotic platforms, with confidence that AL will be improving its predictive performance with each experiment iteration. Furthermore, the study demonstrated that the AL strategy can be applied to an external dataset, thereby widening its applicability. Navigating through an internal dataset achieving 100% accuracy indicates that it has accurately learnt the formulation space, but then it can be employed to predict the processability of a new formulation space. This is in contrast to traditional ML approaches that first require the full dataset to be labelled, which is a time-consuming endeavour. Furthermore, despite hyper-parameter tuning, no previous work has achieved 100% accuracy in predicting printability. Moreover, there are several issues with hyperparameter tuning, including its computationally demanding and, as we previously demonstrated is susceptible to overfitting and does not generalise well to new data [86].

Further work is also needed to identify a metric in gauging whether the learner is improving if AL is to be applied prospectively. Theoretically, the uncertainty could be used. However, as observed herein, the uncertainty was not always reliable, as the uncertainty decreased irrespective of whether the predictions were correct or not. This suggests the models will invariably become more confident, whereas ideally, they should become more confident when they are accurate. Overall, these findings are encouraging and demonstrate that further work into AL in pharmaceutical research is warranted, especially as more sustainable practises are desired [83]. There are different AL strategies to explore, with different querying methods and learners to implement [87]. Furthermore, AL can be applied to predicting numerical values, and future work will seek to develop an AL strategy for predicting the printing temperature needed during 3D printing.

## Conclusion

To obtain a better understanding of AL and its practicality for formulation development, this study applied AL retrospectively to simulate several real-world research scenarios in pharmaceutical 3D printing. Three different 3D printing datasets were tested to comprehensively evaluate AL’s potential, with differences in their data size, component size and printability ratio. Key variables investigated included changing the learner and the starting sample size. Results revealed that a relatively simple AL strategy was effective for the vat polymerisation dataset, which comprised of a balance in printability ratio; whereas AL yielded inconsistent predictions for the FDM and SLS dataset, which were imbalanced. This led to the hypothesis that the learners had a propensity to select the majority class, which in turn hindered AL performance. From the three learners investigated, there was no learner that performed well across all three datasets. This led to a subsequent analysis where the AL model comprised of all three learners and the uncertainties of the minority class were weighted. The analysis revealed that the best strategy was to use ensemble learning with weighting the minority class’s uncertainty by 2-folds. This yielded a balanced accuracy of 100%, 100% and 96.33% for the FDM, vat polymerisation and SLS datasets, respectively. Overall, the study demonstrates how AL can be effectively utilised at the early stages of formulation development, thereby widening the utility of ML in pharmaceutical sciences.

## Supplementary Information

Below is the link to the electronic supplementary material.

Supplementary file1 (DOCX 478 KB)

Supplementary file2 (DOCX 26 KB)

Supplementary file3 (DOCX 486 KB)

## Data Availability

The datasets generated during and/or analysed during the current study are available from the corresponding author on reasonable request.
