# Supplementary material for: Active learning in pharmaceutical 3D printing: a multi-dataset comparison
Source: Drug Deliv Transl Res. 2026 Feb 24;16(10):3872–87. doi: 10.1007/s13346-026-02077-x (PMC13619682; doi:10.1007/s13346-026-02077-x)
Supplement: Supplementary file 1 [file 13346_2026_2077_MOESM1_ESM.docx]

# Active Learning in Pharmaceutical 3D Printing: A Multi-Dataset Comparison

Moe Elbadawi^1^*, Noorul Fathima Abdul Kafoor^1^, Hanxiang Li^2^ and Simon Gaisford^2^

^1^School of Biological and Behavioural Sciences, Queen Mary University of London, Mile End Road, London E1 4DQ, UK.

^2^UCL School of Pharmacy, University College London, 29-39 Brunswick Square, London WC1N 1AX, UK.

* Correspondence: [m.elbadawi@qmul.ac.uk](mailto:m.elbadawi@qmul.ac.uk)

# Section 1: Principles of AL

The aim of subsampling is to investigate a small group of samples to predict the results for a much larger set, avoiding the need to test every single sample. AL is a novel approach to subsampling that uses ML to decide which samples to examine. A recent study showed that AL is more effective than traditional subsampling methods^[1]^.

The AL process is somewhat comparable to the usual ML workflow but operates in a more repetitive, circular fashion. Instead of starting with a large dataset, in AL, you begin with just a small set of data with the goal of exploring and understanding a much larger experimental area. In the context of 3D printing, one starts with a few formulations for which their printability has already been determined. The aim is to use these few formulations to predict the printability of a much larger set of untested formulations. Initially, an ML technique, also known as a learner, is trained on this small dataset and then used to predict the outcomes for the untested formulation. However, unlike traditional ML, AL involves a cycle where, after each round of predictions, a few of the previously untested mixtures are chosen, tested, and then added to the dataset of known formulations. This cycle of predicting, testing, and adding continues, where the predictive accuracy increases with each iteration. In AL, the model simplifies the process by selecting which formulations should be tested next, freeing the researcher from making these decisions. It achieves this by identifying the formulations with the most uncertain predictions, where testing these will provide the most valuable new information for the learner.

Broadly speaking, there are two forms of AL, which are learner- and feature-based AL. For the sake of brevity, we will focus on one learner-based approached, which was implemented in the current study. Learner-based AL uses the learner’s confidence to decide which subset of samples to test. Prior to predicting whether a formulation is printable or not, learners first calculate their probability estimates, with a high probability estimate denoting more confidence. For example, a model with a probability estimate for printable:unprintable of 0.5:0.5 (“fifty-fifty” so to speak) is considered to have a low confidence in its predictions - practically indecisive. Whereas, a probability estimates of 0.9:0.1 or 0.0:1.0 is considered confident for printable and unprintable, respectively. Samples in which the learner is least confident in, or most uncertain in, are selected for testing. This in turn will help a learner learn their characteristics and build its confidence. Once the most uncertain samples have been identified by the learner, the user then tests them and re-trains the learner with the new information. The query cycles continue where ideally with each iteration the learner performance increases, typically until the accuracy convergences to a certain value. The ‘active’ part of AL is the fact that ML actively learns alongside the researcher. Unlike passive ML, AL is more like a human-machine symbiosis, where as both are learning during the process, they both benefit from each other. The human of course benefits by delegating the decision making to the machine, and conversely, the machine benefits by having the human test the queried subsamples. For this reason, AL is also regarded as ‘Human-in-the-loop’ ML.

## Main Components of AL

**Figure S1** illustrates the AL workflow. In learner-based AL, the learner is central to the AL process, as its ability to learn and confidence in its prediction governs key decision making. The learner can be any of the statistical-learning techniques previously utilised, provided that they can predict the probability estimates, including artificial neural networks (ANN), random forest (RF), decision trees and logistic regression (LR). Once trained with the labelled dataset, referred to as ‘labelled’ because each formulation is labelled with a known printability outcome, the learner is then tasked with predicting the outcomes of formulations without labels (the ‘unlabelled’ dataset). For each unlabelled samples, the learner calculates probability estimates to assess its confidence. Samples are then ranked by uncertainty, from high (low confidence) to low (high confidence) uncertainty. The most uncertain samples are selected for labelling by an 'oracle', a term for an external expert or ‘source of truth’, which in the context of 3D printing, refers to a researcher who tests the formulations and records their outcomes. These newly labelled samples are then transferred from the unlabelled dataset to the labelled dataset.

**Figure S1.** Schematic depicting the AL workflow.

In addition to which learner to employ, other considerations include the number of samples to query, the number of overall query cycles, the number of starting samples and the querying strategy. For querying strategy, we employed the least confidence method, which calculates the uncertainty using **Eq (S1)**:

| $s\left( \theta,\boldsymbol{x} \right)=\frac{n\left( 1-{max P}_{\theta}\left( y \vert x \right) \right)}{n-1} \times100$ | **Eq (S1)** |
| --- | --- |

Where *s(θ,x)* is the uncertainty as a function of θ, the model parameters, and *x,* the input data. *n* is the number of predicted classes; $P_{\theta}\left( y | x \right)$ is the probability distribution of *y* given *x*. Essentially, the maximum probability estimate from the probability distribution is determined, normalised and converted into a percentage. For example, the maximum estimate from the distribution of [0.5, 0.5] is 0.5. Subtracting this from 1 equates to 0.5. For binary classes where *n* = 2 (i.e., printable or unprintable), 0.5 is multiplied by 2/(2-1), equating to 1.0. Multiplying this number by 100 gives 100%. Using another example of [0.9, 0.1], the maximum estimate is 0.9 and subtracting it from 1 gives 0.1. Multiplying 0.1 by 2/(2-1) gives 0.2 and multiplying it again by 100 gives 20%. Comparing the two examples, the first has a higher uncertainty and hence would be prioritised.

Other learner-based querying strategies include marginal confidence, entropy, query by committee, which will a subject of future research. Regarding the other considerations, there is no fixed values for how many samples to query per cycle, the total number of cycles nor for the starting number of formulations. It is a testament to the flexibility of AL.

**Reference**

[1]	Y. Wen, Z. Li, Y. Xiang, D. Reker, *Digital Discovery* **2023**, 2, 1134.

# Section 2: Printability Criteria

The following criteria were used to assess whether a literature-extracted formulation was considered printable:

- A formulation was categorised as 'printable' if the literature explicitly described it as such, or used equivalent description;
- A formulation was categorised as 'unprintable' if the literature reported any defects or if images showed deviations from the intended 3D design;
- If a study did not comment on printability but provided details on the formulation's characteristics (e.g., friability, drug release), the formulation was assumed to be printable;
- If a formulation printability status was ambiguous, the study’s authors were emailed for further clarity. If no response received, then the formulations were excluded from the dataset.

# Section 3: MCC Results

**Figure S2**. FDM MCC scores for (A) LR, (B) RF and (C) ANN. The shaded region represents the standard deviation.

**Figure S3**. SLA MCC scores for (A) LR, (B) RF and (C) ANN. The shaded region represents the standard deviation.

**Figure S4**. SLS MCC scores for (A) LR, (B) RF and (C) ANN. The shaded region represents the standard deviation.
