# Supplementary material for: Active learning in pharmaceutical 3D printing: a multi-dataset comparison
Source: Drug Deliv Transl Res. 2026 Feb 24;16(10):3872–87. doi: 10.1007/s13346-026-02077-x (PMC13619682; doi:10.1007/s13346-026-02077-x)
Supplement: Supplementary file 2 [file 13346_2026_2077_MOESM2_ESM.docx]

**Active Learning in Pharmaceutical 3D Printing: A Multi-Dataset Comparison**

Moe Elbadawi^1^*, Noorul Fathima Abdul Kafoor^1^, Hanxiang Li^2^, Chrystalla Protopapa^3^, Marilena Vlachou^3^ and Simon Gaisford^2^

^1^School of Biological and Behavioural Sciences, Queen Mary University of London, Mile End Road, London E1 4DQ, UK.

^2^UCL School of Pharmacy, University College London, 29-39 Brunswick Square, London WC1N 1AX, UK.

^3^School of Pharmacy, Section of Pharmaceutical Technology, National and Kapodistrian University of Athens, 157 84 Athens, Greece.

* Correspondence: [m.elbadawi@qmul.ac.uk](mailto:m.elbadawi@qmul.ac.uk)

**Experimental Procedure for the Validation of Active Learning using Fused Deposition Modelling**

The supplementary document details the experimental protocol for the fused deposition modelling (FDM). For context, the team at university college London (UCL) were interested in determining printable formulations using sulfasalazine, for an internal project. As the sulfasalazine has not been previously 3D printed using FDM, there was no starting point for which excipients to use. Hence, formulation from the literature was used as a starting point [1]. Following the experimental run, the data was used to test the AL model.

**Materials**

The main polymer, Aquasolve® HPMC-AS LG, was purchased from Ashland (USA). Talc and magnesium stearate were purchased from Sigma Aldrich (USA) as filler and lubricant, respectively. Methylparaben was purchased from VWR Life Sciences (USA) to perform the role of plasticizer and sulfasalazine was purchased from Thermo Scientific (USA) as the active pharmaceutical ingredient (API) in the formulation.

**Design of Experiment (DoE)**

A mixture DoE was used to generate the experimental runs for the validation testing. A simplex lattice design with 5 ingredients and a lattice degree of 3, totalling 41 experimental runs. The mixture designed was set such that each experimental run totalled to 1.0 (i.e., 100 w/w%). No additional constraints were added. The mixture DoE was generated using Minitab® v.22.1.

**Hot melt extrusion (HME)**

The DoE-generated formulations were experimentally validated, each of which was mixed to specific composition using a mortar and pestle to ensure a uniform distribution and homogeneity. After mixing, the samples were loaded into a single-screw extruder (Noztek Pro-filament extruder, NozteK, UK) and extruded through nozzles of different diameters at a fixed screw speed of 15 rpm at different temperatures, depending on the specific formulation requirements. In this way, printed filaments suitable for each formulation (if the formulation can be extruded) can be obtained for subsequent printability verification. During the foundation process, HME filament is visually inspected for physical defects (too brittle/ ductile), blackening or surface non-stickiness to approximate extrusion success and printability. During and after extrusion, the diameter of the extruded filament is measured using digital vernier calipers to ensure that the filament diameter is maintained at 1.75mm ± 0.05mm as part of the process quality control.

**Fused Deposition Modelling**

The 3D model of the dosage form was designed using Onshape (PTC Inc., USA) software. The model was subsequently exported as a .stl file so that the geometry selected for the AI-generated formulation in Bambu Studio (Bambu Lab Inc., China) was a tablet with dimensions of X = 10 mm, Y = 10 mm and Z = 3 mm. The printer settings were adjusted accordingly: the fill percentage was 50%, the retraction option was turned off, the extrusion temperature was between 190 °C, the build plate temperature was adjusted to 55 °C, the print speed was kept at 20 mm/s, and the layer height was set to 0.20 mm. The 3D prints were prepared using Bambu X1C (Bambu Lab Inc., China).

**Model Training**

Table 1 represents a sample of the data inputted into the active learning (AL) models. For each of the 41 formulations, the AL model was tasked with predicting their printability, using the ingredients and their respective proportions as inputs. The main manuscripts presents their metrics. If samples were unextrudable via the HME then their printability was recorded as ‘unprintable’.

Table 1. An example of the data generated by the experimental run. The table represents the first ten the DoE sequentially requested. The first five columns pertain to the individual ingredients and their composition values. The remaining three columns refer to aspects of the outcome.

| **Aquasolve LG** | **Methylparaben** | **MgS** | **Talc** | **Sulfasalazine** | **HME Temp** | **Appearance** | **Output** |
| --- | --- | --- | --- | --- | --- | --- | --- |
| 0.666667 | 0 | 0 | 0 | 0.333333 | 70 | Smooth surface with brittle performance | Printable |
| 0.6 | 0.1 | 0.1 | 0.1 | 0.1 | 75 | Rough surface with appropriate printability | Printable |
| 0 | 0.666667 | 0 | 0 | 0.333333 |  |  | Unprintable |
| 0 | 1 | 0 | 0 | 0 |  |  | Unprintable |
| 0 | 0.333333 | 0 | 0 | 0.666667 |  |  | Unprintable |
| 0 | 0.333333 | 0 | 0.333333 | 0.333333 |  |  | Unprintable |
| 0.333333 | 0.666667 | 0 | 0 | 0 |  |  | Unprintable |
| 0.333333 | 0.333333 | 0 | 0.333333 | 0 |  |  | Unprintable |
| 0.666667 | 0 | 0 | 0.333333 | 0 | 63 | White color, smooth surface and good printability | Printable |
| 0.333333 | 0 | 0 | 0 | 0.666667 | 70 | Very rough surface and too brittle to be printed | Unprintable |

**Reference**

[1] Goyanes A, Fernández-Ferreiro A, Majeed A, Gomez-Lado N, Awad A, Luaces-Rodríguez A, et al. PET/CT imaging of 3D printed devices in the gastrointestinal tract of rodents. International Journal of Pharmaceutics. 2018;536:158-64.
