# Supplementary material for: Active learning in pharmaceutical 3D printing: a multi-dataset comparison
Source: Drug Deliv Transl Res. 2026 Feb 24;16(10):3872–87. doi: 10.1007/s13346-026-02077-x (PMC13619682; doi:10.1007/s13346-026-02077-x)
Supplement: Supplementary file 3 [file 13346_2026_2077_MOESM3_ESM.docx]

**Active Learning in Pharmaceutical 3D Printing: A Multi-Dataset Comparison**

Moe Elbadawi^1^*, Noorul Fathima Abdul Kafoor^1^, Hanxiang Li^2^, Chrystalla Protopapa^3^, Marilena Vlachou^3^ and Simon Gaisford^2^

^1^School of Biological and Behavioural Sciences, Queen Mary University of London, Mile End Road, London E1 4DQ, UK.

^2^UCL School of Pharmacy, University College London, 29-39 Brunswick Square, London WC1N 1AX, UK.

^3^School of Pharmacy, Section of Pharmaceutical Technology, National and Kapodistrian University of Athens, 157 84 Athens, Greece.

* Correspondence: [m.elbadawi@qmul.ac.uk](mailto:m.elbadawi@qmul.ac.uk)

**Experimental Procedure for the Validation of Active Learning using Vat Polymerisation**

**Materials**

The active pharmaceutical ingredient (API) buproprion in the form of its hydrochloride salt (BUP⋅HCl) was purchased from Sigma Aldrich Ltd. (Steinheim, Germany). The excipients PEG200, poly(ethylene glycol)diacrylate (PEGDA400), and the photoinitiator phenyl-bis(2,4,6-trimethylbenzoyl)phosphine oxide (TPO) were purchased from the Tokyo Chemical Ltd. (Tokyo, Japan).

**Design of Experiment (DoE)**

A mixture DoE was used to generate the experimental runs for the validation testing. An extreme vertices design with 5 ingredients and a design degree of 1, totalling 25 experimental runs. The mixture designed was set such that each experimental run totalled to 100.0 (i.e., 100 w/w%). Several constraints were added, which were PEGDA400 lower value set to 50.0, BUP⋅HCl with an upper value of 30.0 and TPO with a lower and upper values of 0.1 and 1.0, respectively. This was to reflect that each formulation should contain the core ingredients of a photopolymer (i.e., PEGDA400) and photoinitiator (TPO), and from prior experience, the API-loading should not exceed 30 w/w%, at which amount the admixture becomes unprintable. The mixture DoE was generated using Minitab® v.22.1.

**Vat Polymerisation**

All ingredients were precisely weighed using an ATX224® high-precision analytical balance (Shimadzu, Kyoto, Japan). PEGDA used as the photopolymerizable monomer, while TPO served as photoinitiator. Each formulation, proposed by DoE (Table 1), was prepared by dissolving BUP⋅HCl in water under continuous stirring at 60 °C using a magnetic stirrer (PCE-MSR 450, Manchester, UK). In formulations with a high drug concentration, the mixture was sonicated in an LBS 2 Ultrasonic bath (4.5 L, 285W, 59 KHz) (FALC INSTRUMENTS, Treviglio, Italy) at 45 °C to enhance its solubility. Subsequently, the rest of the excipients were added, and the mixture was stirred for an additional 10 min to ensure homogeneity. TPO was then added under stirring for approximately 2 hours or until fully solubilized. To prevent premature polymerization and solidification, the mixture was shielded from UV exposure, using a protective cover.

The produced formulation was poured onto a resin tray, to be printed. All tablets were fabricated using a Liquid Crystal Display (LCD) 3D printer (Halot Sky, Creality, Shenzhen, China), equipped with a 405 nm laser capable of achieving a resolution of 0.034 mm, a layer thickness between 0.01–0.2 mm, and a printing speed of 1–4 seconds per layer (Table 2). The same printing parameters were used for all formulations to ensure objectivity and consistency to the printability. The tablet dimensions (8 mm × 8 mm × 3 mm) were set using the 3D Builder software and saved in stereolithographic (.stl) format. Printing was conducted directly on the build platform, at ambient temperature, without any additional supports.

After printing, the tablets were rinsed with deionized water, cautiously dried with a filter paper to remove uncured material and exposed to UV light for 2 more min to ensure complete polymerization.

| **Formulations** | **PEGDA400 (%)** | **PEG200 (%)** | **H_2_O (%)** | **BUP⋅HCl (%)** | **TPO (%)** |
| --- | --- | --- | --- | --- | --- |
| 1 | 55.7 | 30.2 | 5.7 | 7.5 | 0.8 |
| 2 | 80.2 | 5.7 | 5.7 | 7.5 | 0.8 |
| 3 | 50.0 | 49.9 | 0.0 | 0.0 | 0.1 |
| 4 | 50.0 | 49.0 | 0.0 | 0.0 | 1.0 |
| 5 | 50.0 | 0.0 | 19.0 | 30.0 | 1.0 |
| 6 | 55.7 | 5.7 | 30.7 | 7.5 | 0.3 |
| 7 | 55.7 | 5.7 | 30.2 | 7.5 | 0.8 |
| 8 | 55.7 | 5.7 | 15.7 | 22.5 | 0.3 |
| 9 | 55.7 | 15.2 | 5.7 | 22.5 | 0.8 |
| 10 | 50.0 | 19.0 | 0.0 | 30.0 | 1.0 |
| 11 | 55.7 | 15.7 | 5.7 | 22.5 | 0.3 |
| 12 | 50.0 | 0.0 | 49.0 | 0.0 | 1.0 |
| 13 | 55.7 | 5.7 | 15.2 | 22.5 | 0.8 |
| 14 | 80.7 | 5.7 | 5.7 | 7.5 | 0.3 |
| 15 | 65.2 | 5.7 | 5.7 | 22.5 | 0.8 |
| 16 | 99.9 | 0.0 | 0.0 | 0.0 | 0.1 |
| 17 | 65.7 | 5.7 | 5.7 | 22.5 | 0.3 |
| 18 | 69.0 | 0.0 | 0.0 | 30.0 | 1.0 |
| 19 | 50.0 | 0.0 | 49.9 | 0.0 | 0.1 |
| 20 | 69.9 | 0.0 | 0.0 | 30.0 | 0.1 |
| 21 | 99.0 | 0.0 | 0.0 | 0.0 | 1.0 |
| 22 | 50.0 | 0.0 | 19.9 | 30.0 | 0.1 |
| 23 | 61.5 | 11.5 | 11.5 | 15.0 | 0.6 |
| 24 | 50.0 | 19.9 | 0.0 | 30.0 | 0.1 |
| 25 | 55.7 | 30.7 | 5.7 | 7.5 | 0.3 |

Table 1: The formulations suggested by the DoE

Table 2: The printing parameters

| **Parameters** | **Values** |
| --- | --- |
| Layer thickness | 0.050 mm |
| Initial exposure | 40 s |
| Exposure time | 3 s |
| Rising high | 8 mm |
| Motor speed | 5 mm/s |
| Turn off delay | 4 s |
| Bottom exposure layer | 2 |

**Results/Discussion:**

The printability of each formulation is shown in Table 3, and the printed tablets of the printable formulations are displayed in Figure 1A. Some formulations were not printable, due to two main reasons. First, although the drug is hydrophilic, its high concentration, in the formulations proposed percentage, leads to a saturated solution. Second, while PEGDA400 is relatively soluble in water, in some cases, the PEGDA400/H₂O concentration ratio resulted in an immiscible mixture, forming a turbid solution (Figure 1B).

Interestingly, in certain cases, in the absence of the drug, turbidity was noticed, when the concentration of water in the PEGDA400-H₂O mixture was high. This, however, changed upon the addition of BUP⋅HCl, in the formulations, leading to clear and printable solutions. This is because a part of the requisite amount of water in preparing the aforementioned formulations is trapped in the form of water of crystallization in the process of dissolving BUP⋅HCl. The reduced amount of water left in these formulations is optimal, resulting to a clear solution.

Table 3: The printability of each proposed formulation

| **Formulations** | **Formulation uniformity** | **Outcome (YES/NO)** |
| --- | --- | --- |
| 1 | Yes | Yes |
| 2 | Yes | Yes |
| 3 | Yes | Yes |
| 4 | Yes | Yes |
| 5 | Yes | Yes |
| 6 | Yes | Yes |
| 7 | Yes | Yes |
| 8 | Yes | Yes |
| 9 | No (the drug is not dissolving in the formulation) | No |
| 10 | No (the drug is not dissolving in the formulation) | No |
| 11 | No (the drug is not dissolving in the formulation) | No |
| 12 | No (The PEGDA400 in that amount was not mixable with water, turbid solution) | No |
| 13 | Yes | Yes |
| 14 | Yes | Yes |
| 15 | No (the drug is not dissolving in the formulation) | No |
| 16 | Yes | Yes |
| 17 | No (the drug is not dissolving in the formulation) | No |
| 18 | No (the drug is not dissolving in the formulation) | No |
| 19 | No (The PEGDA400 in that amount was not mixable with water, turbid solution) | No |
| 20 | No (the drug is not dissolving in the formulation) | No |
| 21 | Yes | Yes |
| 22 | Yes | Yes |
| 23 | Yes |  |
| 24 | No (the drug is not dissolving in the formulation) | No |
| 25 | Yes | Yes |

**(A)**

**(B)**

**Figure 1:** (A) The tablets developed by the LCD 3D printer of the printable formulations (B) the turbid solution of the unprintable formulations
